# Fex-Talk: a Short Educational Intervention Intended to Enhance Nurses’ Readiness to Discuss Fertility and Sexuality with Cancer Patients

**DOI:** 10.1007/s13187-019-01493-7

**Published:** 2019-03-01

**Authors:** Jeanette Winterling, Claudia Lampic, Lena Wettergren

**Affiliations:** 1 Department of Neurobiology, Care Sciences and Society, Karolinska Institutet, 23 300, SE-141 83 Huddinge, Sweden; 2 Patient Area of Hematology, Karolinska University Hospital, Stockholm, Sweden; 3 Department of Women’s and Children’s Health, Karolinska Institutet, Stockholm, Sweden

**Keywords:** Education, Nurses, Cancer, Communication, Fertility, Sexuality, Young adults

## Abstract

Sexual and reproductive health is known to generally be insufficiently addressed by health care personnel working in cancer care. We hence developed a short educational intervention, Fex-Talk, to overcome the barriers to communicate about sexuality and fertility. The present study sought to evaluate the Fex-Talk intervention, which aims to enhance nurses’ readiness to discuss fertility and sexuality issues with cancer patients. The educational intervention involves a single session with an optional follow-up session, and it includes different components in accordance with Kolb’s experiential learning cycle. The evaluation was based on participants’ oral and written feedback regarding the content and organization of the intervention, as well as on teachers’ field notes from five educational events involving nurses who work with cancer patients (*n* = 140). The data were analyzed using a thematic approach. Four themes were identified, namely increased awareness, need for knowledge, challenging discomfort, and dealing with external obstacles. The intervention increased participants’ awareness of patients’ need to discuss sexuality and fertility and of their own need for additional knowledge. The role-play exercise was said to challenge personal discomfort, although the participants still felt it helped to boost their courage to, in the future, engage in such conversations. Several external obstacles to initiate a conversation about sexuality or fertility were identified, and possible strategies for overcoming them were discussed. In conclusion, the Fex-Talk intervention was experienced positively by the participating nurses. The results indicate that the intervention increased nurses’ understanding of patients’ needs related to sex and fertility and overcome barriers to initiate discussions about sex and fertility with patients.

## Introduction

An increasing number of individuals are now being cured of their cancer or are receiving treatment that can result in many years of survival even when the disease is incurable. The key goal for these individuals is to enjoy a meaningful existence and a high quality of life, which encompasses their need for sexual and reproductive health. However, sexual problems (e.g., vaginal dryness and erectile dysfunction) and decreased fertility are both common sequelae of cancer and the associated treatment [1]. Previous research has shown that cancer patients would like health care personnel to raise these issues and to provide them with information and advice [2–4]. Yet, many patients still find that the care provided to them in this regard is insufficient or lacking [3, 5–7].

Nurses working in the field of oncology care engage in frequent patient encounters, and they are hence highly suitable candidates for addressing issues related to sexuality and fertility. Although oncology nurses recognize that addressing patients’ sexual health [8–10] and reproductive concerns [11] is an important aspect of nursing, they often avoid or fail to effectively respond to patients’ needs for communication regarding such matters [8–11]. Nurses and other health care professionals typically perceive a number of barriers to the initiation of conversations about sexuality and fertility matters with patients, including a fear of causing patients distress or discomfort [3, 8, 12], a lack of knowledge about such issues [8, 12, 13], their own feelings of personal discomfort, or insecurity [3, 9], as well as practical/organizational issues that limit the opportunities for private conversations [3, 8, 9, 12]. Indeed, several prior studies have suggested that further education and training are needed to overcome these barriers within cancer care [4, 7, 9, 10, 13]. Health care professionals often believe that patients want information presented to them in a medicalized fashion, although research has indicated that patients would prefer help with a broad array of sexual and fertility issues [3]. The PLISSIT model has been widely used by health care providers to address needs related to sexual well-being in individuals with physical conditions [22]. The model describes four increasing levels of involvement in order to help practitioners identify their role in assessing patients’ sexual problems, namely giving permission (P), providing limited information (LI), specific suggestions (SS), and intensive therapy (IT). It has been suggested that the first step, that is, the permission level, is often overlooked by practitioners, which is why an extended version, the Ex-PLISSIT model, has been proposed [22]. The Ex-PLISSIT model extends the original model by emphasizing the role that giving permission plays during all the stages, as well as how each stage is underpinned by the concept of giving permission [22].

Nurses working in the field of oncological care are suggested to have the potential to meet patients’ needs of information and dialog around sex and fertility, so long as they are offered relevant training. Although nurses are trained in general counseling, specific training in communication regarding sexuality and fertility is seldom included in the nursing curriculum. The few educational interventions aimed at health care professionals working within oncological care that are described in the literature focus on either sexual [14, 15] or fertility issues [16, 17], and they vary considerably in terms of their length, from brief (30–45 min) training sessions [15] to a 2-year program [14]. As both sexuality and fertility matters are sensitive issues that often coexist in patients within the fertile age range, we developed a short educational intervention intended to overcome the identified barriers to initiate related conversations with patients. The present study evaluates that intervention, which aims to enhance nurses’ readiness to discuss fertility and sexuality issues with cancer patients.

## Methods

### Development of the Intervention

The present study was conducted as part of the Fertility and Sexuality Following Cancer (Fex-Can) research program, which investigates and treats sexual problems and fertility-related distress in young adults with cancer [18, 19]. The educational intervention, which is known as Fex-Talk, was developed to be a part of courses given for nurses at advanced levels. The content of the intervention was based on our prior research, a review of the literature, clinical practice, and experiences from nursing education. Kolb’s experiential learning cycle, which is defined as “the process whereby knowledge is created through the transformation of experiences and knowledge results from the combination of grasping and transforming experience” (p. 41), was used as a pedagogical model [20]. The learning outcomes for the intervention were to reflect on patients’ perspectives on sexuality and fertility in relation to having cancer and to dare initiate conversations about sex and fertility with patients.

### Structure of the Intervention

**Fig. 1:**
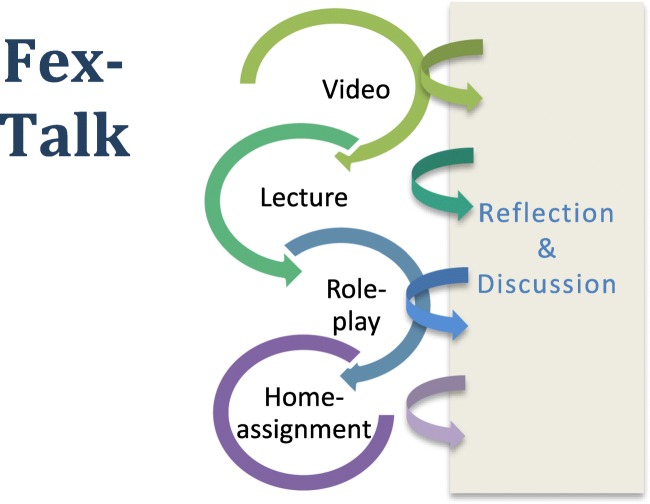
Outline of the components included in the intervention

The intervention was specifically developed to involve only a single session lasting approximately 2 h. If the course schedule permitted the Fex-Talk also included a follow-up session. In order to activate learning through experience, different components were included in the intervention (see Fig. 1).

#### Video

To provide the participants with insights into how adolescent and young adult patients and their partners may experience difficulties related to sex and intimacy, a 13-min-long video of a panel discussion organized by the Swedish patient organization “Young Cancer” was shown. Afterwards, the participants were encouraged to reflect on their own clinical experiences of interactions with patients.

#### Lecture

To provide the participants with basic facts about the impact of cancer on both sexuality and fertility, a short lecture was given.

#### Role-Play

To increase the participants’ self-confidence regarding raising sensitive issues with patients, they were engaged in role-plays concerning the initiation of conversations about fertility and sexuality. As an introduction, the teachers performed an improvised role-play depicting a nurse who initiated a conversation with a patient. Thereafter, the participants were divided into pairs and provided with three short scenarios to role-play, which illustrated common situations encountered in cancer care. They were also able to create their own scenarios based on their clinical experience. The participants were provided with examples of sentences that could be used to start a conversation with a patient, including “It is not uncommon for cancer and cancer treatment to affect a person’s sex life and relationships, at least for a while. How has this been for you? Is it something you’ve been thinking about” and “Cancer treatment may affect the ability to have children. Has anyone talked to you about this?” “Is it something you’ve been thinking about?” During role-plays, the participants took turns to play the nurse/patient. Finally, the whole group was gathered together and asked about their experiences of the role-play exercise to encourage reflection.

#### Homework Assignment

Where possible, a follow-up session was held 3 to 4 weeks after the initial session. In preparation for the follow-up session, the participants were given a homework assignment that required them to practice initiating conversations about fertility and/or sexuality with three patients at their current workplace. The participants were instructed to document their experiences in field notes using a specific form that they were provided with. During the follow-up session, the participants were divided in smaller groups, with one teacher per group, and they discussed their experiences based on the field notes. The small-group discussions were then summarized for the whole group and the take home messages were agreed upon.

### Evaluation of the Intervention

The Fex-Talk intervention was evaluated after it had been performed with five groups of nurses during the period 2016 to 2018 (total *n* = 140). The intervention was delivered as part of a master’s level study program in specialist nursing in oncology care (two occasions, each including a follow-up session), a separate course in oncological care (one session), a 1-day course for nurse navigators in cancer care (one session), and a course in pediatric oncology care for nurses (one session). The evaluation was based on the participants’ written and/or oral feedback regarding the content and the different components of the Fex-Talk, which was gathered directly after each session, as well as on the teachers’ observations and field notes from each session. The data (194 written comments from the participants and 46 specific field notes from the teachers) were analyzed using a thematic approach [21]. The first author started with familiarization of data and generated initial codes that were sorted into potential themes, which were reviewed, defined, and named in collaboration with all authors. As all authors had been involved in delivering the educational intervention, a credibility check of the derived themes was performed by a person with no involvement in the Fex-Talk. This person, a psychologist with some experience of qualitative research methods, was provided with an unsorted list of the written comments/field notes and requested to sort these into the four key themes. This sorting largely confirmed the original themes and consensus was achieved following discussion of specific comments.

## Results

Four key themes were identified in the data, namely increased awareness, need for knowledge, challenging discomfort, and dealing with external obstacles. These themes are elaborated on below.

### Increased Awareness

Many participants reported that the Fex-Talk intervention increased their awareness that sexuality and fertility are both important issues for cancer patients. The participants thought that the video in which several patients and one spouse related their own stories was a valuable means of introducing the subject. It served as an eye-opener for the participants due to presenting the current situation concerning sexuality and fertility issues in cancer care. As one student commented after watching the segment of the video in which the partner of a patient with leukemia shared her experience of a lack of relevant information about physical contact during chemotherapy from health care providers:

“Not daring to hug your husband, now that’s truly the result of care that does not prioritize sexuality. I hadn’t considered it before.”

In the written evaluation, many participants described that the Fex-Talk intervention suggested ways in which current cancer care is lacking or could be improved. The participants felt that they had come to understand how important these issues are for patients, and furthermore, they acknowledged that such subjects are not addressed sufficiently in current clinical practice.

“The video was good. It was interesting to hear how those who are affected by cancer experienced the information or, rather, the lack of it.”

However, a few participants commented that they were already aware of the importance of these issues in relation to cancer care.

The teachers reported that, after watching the video, the participants became engaged in lively discussions about how important issues of sexuality and fertility are for patients and their partners, as well as why such issues are seldom raised.

### Need for Knowledge

Many participants expressed, both in their written comments and during the intervention, a need for more knowledge to better understand the potential sexual problems and fertility impairments related to cancer. The participants believed that such knowledge would make them more confident to raise the subject. A desire for more information about physiology and anatomy related to sexual and fertility problems was emphasized, as was a wish for facts regarding how cancer and its treatment affect patients.

“I wish to know more facts about what actually happens to patients, as well as what I should say to them.”

While many participants expressed the need for more knowledge, a significant proportion of them also noted how the short lecture presented new or deeper facts concerning the impact of cancer and cancer treatments on sexuality and fertility. Some participants emphasized that they had learned they did not need extensive knowledge about sexuality and fertility in relation to cancer in order to be able to raise such issues with patients.

“Just making it simple and addressing the issue of sexuality/fertility makes a big difference, even though you don’t always have all the answers.”

The combination of different components used in the intervention fostered the participants’ sense of active participation, and words such as “inspiring” and “interesting” were used when describing their impressions of the intervention.

“I think it was good, since it offered a lot of knowledge about the subject and from different angles, too. I really appreciated the active discussions.”

The participants reported appreciating the many opportunities to raise and debate specific questions, for example, the risk of transmitting chemotherapy drugs during intimate contact between patients and their significant others, suitable options for contraception, and whether patients may have intercourse when they are neutropenic. Some participants stated that they wanted more facts than were provided. The participants who worked with children and adolescents expressed that such issues are especially difficult, and they wished that the intervention had addressed this specific area further.

The teachers reported that the amount and level of facts included in the Fex-Talk appeared to be enough for initiating role-plays. However, if more scheduled time would be available for the educational intervention, the teachers recommended an extended lecture including reasons for sexual dysfunction and impaired fertile ability due to cancer and its treatment, information, and advice for sexual practice and fertility care.

### Challenging Discomfort

Many students reported that the Fex-Talk intervention helped them to challenge their own discomfort when it comes to communicate about such sensitive issues. It was commonly felt by the participants that they need to have the courage to initiate conversations about sexuality and fertility with patients, even if they feel uneasy about it. One participant wrote:

“I learned not to ignore these issues because they seem uncomfortable. I must dare to ask questions and find opportunities to raise questions about sexuality and fertility… We must become better and dare to take such a step!”

Many participants described how it was useful and instructive to practice initiating a conversation about sexuality and fertility with a patient through role-plays, as well as to discuss and reflect on the exercise afterwards. They believed it was useful to practice something they perceived to be difficult, and they thought the exercise would make it easier to initiate such discussions in the course of their clinical work. Some participants thought it was fun to participate in the role-play, although others reported that it was difficult and challenging. As one participant put it:

“The role-playing was difficult, perhaps because it felt artificial, although it was still good to try to say the different sentences and find the best wording.”

Even though all the participants took part in the role-plays, some of them expressed negative comments about the exercise. Some felt very uncomfortable talking about sexuality and/or fertility, and they described it as embarrassing. Others expressed how they did not appreciate engaging in role-plays as such and felt that they lacked enough imagination to develop the role-played situation. It was suggested that more detailed information about the different scenarios would have proved helpful, since it might have facilitated understanding the roles they were about to play. Other participants would have preferred discussions regarding how best to communicate about sexuality and fertility with patients, without the need to engage in role-plays. However, the teachers thought that the role-play exercise worked well in all the groups, since the participants actively engaged in the process and no one refused to participate.

According to teacher’s field notes, the participants raised several clinical situations that were perceived as challenging. For example, situations with very young patients or a large age difference between the patient and his or her partner were perceived as challenging to handle when discussing sex as well as fertility.

### Dealing with External Obstacles

The participants described several external obstacles to initiate conversations about sexuality and/or fertility, and strategies for overcoming the identified obstacles were also discussed. The obstacles were related to the environment and organization of care, and they included lack of time, routines, and privacy.

Discussions about the obstacles were common directly following the role-plays, as well as during the follow-up sessions, when it became evident that many participants had not been able to complete their homework assignments. The participants who had not done their homework often described their reasons for failing to do so based on external obstacles, such as a lack of time or single rooms, the presence of a significant other (e.g., partner or parent), or a lack of routines for raising such issues with patients. Some participants felt that communicating about sex and fertility was not part of their usual practice, and they were uncertain about when and how to do so. They did, however, share experiences and ideas concerning how to deal with different obstacles.

“It’s great to hear other people’s thoughts and reflections. It prompts you to think about how to better address these questions.”

The suggested strategies for overcoming the obstacles included adding sexuality and fertility to existing checklists, using written materials containing information compiled for the specific patient groups being treated at their workplace, and the possibility of creating diagnosis-specific informational material.

The teachers’ field notes included descriptions of situations when patients had incorrect or contradictory information from different health care providers which was perceived as difficult. Also, some participants expressed that they perceived resistance to include these issues in their organization and a lack of sufficient resources to accommodate patients’ needs.

## Discussion

The Fex-Talk intervention with its components seems to be a suitable package and the results indicate that the intervention increased nurses’ awareness of the importance of addressing sexuality and fertility when caring for young cancer patients. Furthermore, the educational intervention appeared to boost the participants’ confidence to, in the future, initiate discussions with patients regarding such topics.

A common barrier reported by health care personnel is the fear of causing patients distress or discomfort [3, 8, 12]. We hence believe that the first part of the Fex-Talk session, when we show video featuring patients and their partners who share their experiences of how sexual issues have been addressed in their care, is crucial. It is concrete and illustrates the reality of most patients’ situations, and it corresponds to the first step in Kolb’s experimental cycle [20]. Most participants highlighted how the video made them realize that patients and their relatives want health care personnel to raise sexuality and fertility issues, regardless of whether the patient is receiving curative treatment or palliative care. The overall evaluation of the Fex-Talk intervention shows that the participants’ developed an enhanced awareness for how important sexuality and fertility issues are for patients, although it is unclear if their sense of responsibility likewise increased.

A lack of knowledge is commonly described as a barrier for nurses in terms of raising issues about sexuality and fertility in cancer care [8, 12, 13]. However, we question whether nearly receiving facts will help nurses to overcome such a barrier. Some participants did express the desire for more facts in the Fex-Talk intervention, and they argued that additional information would make it easier to start chatting with patients about these topics. However, most of the situations the participants described from their clinical practice of initiating discussions about a patient’s sex life or fertility did not reflect a need for facts, since the patients typically raised concerns related to a partner. This lends support to our decision to focus more on giving patients permission to talk about such issues, as suggested in the Ex-PLISSIT model [22]. By giving a person explicit permission to discuss any concerns they may have about their sexuality, that person is affirmed as a sexual being, while any information or suggestions that follow are then specifically tailored to the needs of that person [22]. As the session was only 2 h long, the provision of more information would have prevented the focus on teaching participants to give their patients permission to talk about these issues.

One of the most difficult barriers to overcome in terms of discussing issues about sexuality and fertility in cancer care is the individual health care worker’s own sense of discomfort, insecurity, or shyness [3, 9]. The video used at the beginning of the Fex-Talk intervention did “desensitize” the participants to the embarrassment and sensitivities associated with discussing such issues, which has previously been reported in relation to another educational intervention about sexuality for medical students [23]. In order to overcome the participants’ own discomfort, we used a role-play approach because it embodies the key elements of experimental learning [20]. As prior studies have reported that participants may experience humiliation and embarrassment when performing role-plays [24], we tried to lower the expectations for the performance, as well as to minimize the presumed discomfort for the participants, by having the teachers perform an improvised role-play at the beginning, suggesting a clinical situation they were familiar with, providing sentences they could start with, and dividing them into pairs with no observer [24]. In the present study, some participants described how the role-plays helped them to overcome their feelings of discomfort, while others seemed to have a better experience doing the homework assignment. Some participants felt very uneasy about the role-plays. One way to decrease this sense of unease further would be to use simulated patients, who would be played by expertly trained actors skilled in improvisation techniques [24], although this might not help much as the subject matter itself is difficult for many people to talk about and may evoke strong feelings.

Several studies have reported that external aspects, such as a lack of time and private space, actually limit the opportunities for conversations about sexuality and fertility issues in cancer care [3, 8, 9, 12]. For example, in a similar intervention about sexual health involving two workshops, more than half of the participating nurses reported not having enough time to discuss sexuality, while half of them perceived that it was the working environment that prevented them from doing so [14]. Such barriers were also referenced several times by participants in the discussions included in the Fex-Talk intervention, especially when they reported why they had been unable to complete their homework assignment between sessions one and two. Those participants who took part in the Fex-Talk intervention with a follow-up session had the opportunity to hear other participants’ experiences of being able, or being unable, to initiate conversations with patients. In the discussions following the homework assignment, the participants again reported how listening to their fellow participants’ experiences increased their learning opportunities, as described in Kolb’s experiential learning cycle. We hence recommend that the Fex-Talk intervention includes two sessions, rather than just one.

The Fex-Talk intervention appeared to increase nurses “awareness and readiness to discuss sexuality and fertility issues with their patients. However, if these new insights and skills are sufficient to change nurses” actual behavior during encounters with patients was not assessed in the present study. An evaluation of a similar short educational program (A 4-h session including lectures) regarding fertility preservation for health care providers working in oncology care revealed that participants’ knowledge and confidence increased, although their self-practice did not change [17]. Yet, an evaluation of another intervention (two 5-h workshops including short lectures, group discussions, and role-play) showed that those who attended two workshops reported engaging in discussions about sexual issues with their patients more often than those who did not attend [14].

Overall, the different components of the intervention (video, lecture, role-play, homework assignment, reflection, and discussion) appeared suitable, since all the participants actively took part in all of them. In their feedback, the participants reported that they liked the combination of different components, and especially that the reflections and discussions were important. As described in Kolb’s theory, both reflective observation and abstract conceptualization enhance the experiential learning cycle [20]. In order to further develop the Fex-Talk intervention, more teaching materials could be included, which was found to be useful in a prior study [24]. One suggestion is to develop a pocket guide for staff that includes sentences intended to aid in initiating communication with patients about sexuality- and fertility-related issues and to conduct a basic assessment in this regard [14]. Another suggestion is to provide participants with more knowledge about a network of specialists they could refer to, although it is important to remember that there is a danger here of shifting responsibility onto someone else [3]. Other professions, such as medical doctors and social workers, could also benefit from this intervention. The Fex-Can program was developed specifically for cancer care, but sexuality and fertility issues are rarely raised in other care settings, so we have started to develop the same educational intervention for nurses working in other areas, such as in medicine, surgery, and children. The key element is to provide examples that are related to the area of each nurse’s clinical work.

## Conclusion

Overall, the Fex-Talk intervention was experienced positively by the participating nurses. The results indicate that the intervention increased nurses’ understanding of patients’ needs related to sex and fertility and overcome barriers to initiate discussions about sex and fertility with patients.
